# A polyphasic method for the characterization of epiphytic diatoms growing on *Gelidium corneum*

**DOI:** 10.1016/j.mex.2025.103188

**Published:** 2025-01-28

**Authors:** María Borrego-Ramos, Raquel Viso, Saúl Blanco, Begoña Sánchez-Astráin, Camino F. de la Hoz, José A. Juanes

**Affiliations:** a Diatom Lab, IMA, La Serna St., León, Spain; b Department of Ecology, Faculty of Science, Charles University, Viničná 7, Prague 2, CZ-12844, Czech Republic; c Ecology Unit, Department of Biodiversity and Environmental Management, Faculty of Biological and Environmental Sciences, University of León, León, Spain; d IHCantabria - Instituto de Hidráulica Ambiental de la Universidad de Cantabria, Santander, Spain

**Keywords:** Protocol, Bentos, Epiphytism, Macroalgae, Bacillariophyta, *RBCL marker*, Metabarcoding, A polyphasic method for the characterization of epiphytic diatoms growing on *Gelidium corneum*

## Abstract

Epiphytic diatoms associated with marine macroalgae play vital ecological roles in nutrient cycling and primary production, yet their study remains limited due to the lack of standardized methodologies. This study focuses on diatom communities growing on *Gelidium corneum*, a key red alga in the Cantabrian coast (Spain). Samples were collected from two depths along the northern coast of Spain and processed using both morphological and molecular approaches. Morphological analysis involved diatom frustule preparation using hydrogen peroxide digestion, acid treatments, and permanent slide mounting, enabling identification through light microscopy. Molecular analysis employed DNA extraction and *rbcL* marker-based metabarcoding, allowing detailed taxonomic characterization. Results highlight the efficacy of combining morphological and molecular techniques to overcome the limitations of either approach individually. By standardizing procedures, we enhance the reproducibility and comparability of studies focused on diatom epiphytes. Our results highlight the ecological significance of diatom-macroalgal interactions and provide a framework for future investigations into these essential but underexplored communities.

• A polyphasic method was developed for studying epiphytic diatoms on *Gelidium corneum*, combining morphological and molecular tools.
• The approach overcomes challenges in diatom characterization, including intricate host morphology and cryptic species identification.
• Standardized protocols enhance reproducibility and offer insights into diatom-macroalgal ecological interactions.

## Graphical abstract

**Figure:**
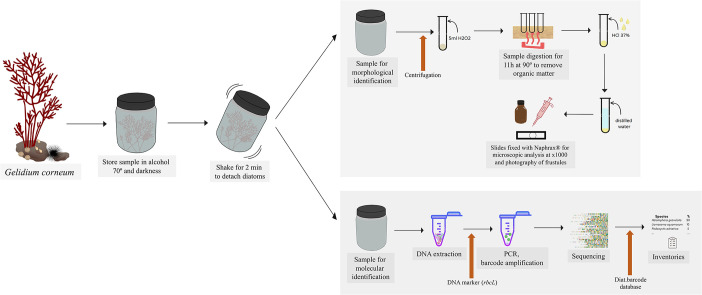

Specifications table

**Table:** 

Subject area:	Environmental Science
More specific subject area:	Algal biofilms
Name of your method:	A polyphasic method for the characterization of epiphytic diatoms growing on *Gelidium corneum*
Name and reference of original method:	None
Resource availability:	Software: Diat.barcode package in R software Data: DNA reference library (Diat.barcode, available at https://carrtel-collection.hub.inrae.fr/barcoding-databases/diat.barcode

## Background

Microorganisms, which include bacteria, archaea, fungi, microalgae, protists, and marine viruses, constitute the majority of marine biomass and play pivotal roles in primary productivity and ecosystem functions. These functions include organic matter decomposition [1] and the cycling of carbon and nitrogen [2,3]. Marine microbes inhabit water columns and colonize substrates such as rocks, sediments, plants, and animals [4]. Living substrates provide suitable habitats, often fostering symbiotic relationships that enhance host fitness through functions like nitrogen fixation [5,6], morphogenesis induction [7], and chemical defense [8,9].

Despite extensive research on terrestrial plant microbiomes and marine invertebrate symbioses [[10], [11], [12]], macroalgae microbiomes remain underexplored, though their ecological importance is gaining recognition. Seaweeds are vital to coastal ecosystems, offering food, shelter, and biodiversity-rich habitats while contributing to primary production and nutrient cycling [13,14]. Seaweed surfaces release compounds that shape host-microbe and microbe-microbe interactions [15]. Studies on seaweed-bacteria interactions reveal their role in nutrient uptake and host defense [16,17].

Epiphytic diatoms associated with seaweeds significantly contribute to primary production, accounting for 10% to 50% of the total photosynthetic rate of the host-epiphyte community [18]. Given that diatoms are responsible for at least 40% of oceanic primary production, their ecological role enhance ecosystem productivity and support energy flow and ecological dynamics [18,1,19]. While most studies have focused on epiphytes in seagrasses [[20], [21], [22]], research on epiphytic microalgae in seaweeds remains limited. Indeed, recent research on epiphytic diatom communities has revealed diverse species on macroalgae worldwide [[23], [24], [25]], with notable variations in diatom abundance across different morphological and functional regions of the seaweed thallus [26,27]. Thus, linking diatom taxa to their functional roles in macroalgal ecosystems could offer valuable insights for management, highlighting the need for more studies to understand the ecological implications of these associations.

*Gelidium corneum* (Hudson) J.V. Lamouroux, 1813, is a red seaweed that forms extensive meadows in sublittoral communities along the coast of the Cantabrian Sea [28,29], with its distribution extending across the North Atlantic, from France to Morocco. As a canopy-forming species, it creates a complex and stable environment that provides habitat and shelter for various organisms, including fish, invertebrates, and other algae [30]. During the summer, *G. corneum* becomes colonized by a range of epiphytes, while in winter, increased wave action and branch loss result in a reduced epiphyte load [31]. Key epiphytes include macroalgae (e. g., *Plocamium cartilagineum, Dictyota dichotoma*) and bryozoans (e. g., *Electra pilosa, Scrupocellaria* sp*.*), which are identified as the main epibionts [[32], [33], [34]]. However, data on the contribution of microorganisms to these associations remains poorly understood.

Epiphytic diatoms, essential components of the epiphyton, are influenced by factors like environmental stress, host structure, and age, and physico-chemical parameters significantly shape their communities [35]. Despite their importance, standardized methods to analyze these assemblages are lacking.

This study presents a methodological protocol for characterizing the epiphytic diatom assemblage on *G. corneum*, addressing quantitative and qualitative aspects. The protocol, adaptable to other macrophyte-microbial systems, aims to standardize approaches for studying these ecologically significant communities.

## Method details

**Fig. 1:**
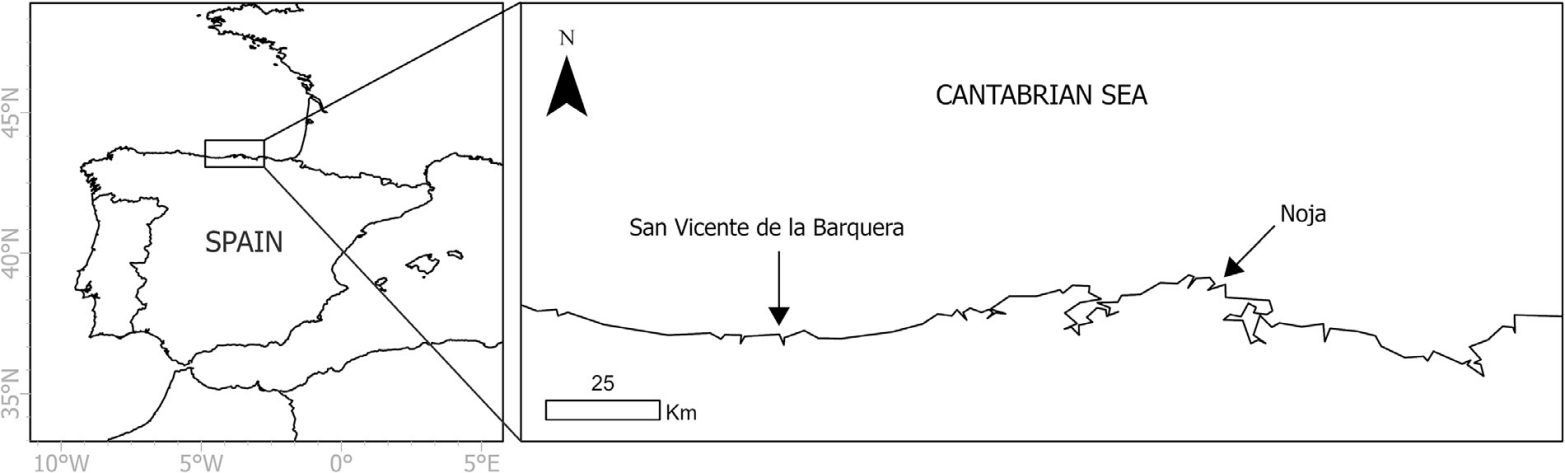
Map of the sampling sites of the study on the north coast of Spain: San Vicente de la Barquera and Noja.

This method was tested on samples collected from two locations along the northern coast of Spain, specifically within the Cantabria region: near San Vicente de la Barquera and Noja (43° 22′ 59″ N, 4° 24′ 01″ W and 43° 28′ 53″ N, 3° 31′ 07″ W, respectively), during June 2022 (Fig. 1). The Cantabrian coast features a temperate-humid climate influenced by Atlantic winds predominantly coming from the W-NW, although under anticyclonic conditions NE winds dominate [36]. Mean seawater temperature along this coastline typically range from approximately 12 °C in winter to 20 °C in summer, reflecting the moderating effect of the oceanic currents [37].

Depths of 5 and 15 m were chosen at both locations (San Vicente de la Barquera and Noja) for the specimen collection, corresponding to a typical habitat range of this species. Thus, these conditions were selected to ensure variation in light conditions. Healthy and robustly developed specimens of *G. corneum* were collected through scuba diving, cutting them from the base to ensure their future recovery. Then specimens were carefully introduced into containers of 1 L of capacity. Upon reaching the surface, seawater was decanted from the containers, and 70% alcohol was added until the samples were fully submerged. Subsequently, the containers were stored inside cool boxes and transported to the laboratory. Upon arrival samples were maintained at 4 °C in darkness until processing.

### Pre-treatment

Each sample was shaken for two minutes to remove diatoms, in accordance with previous studies on diatoms associated with macrophytes [[38], [39], [40]]. The algae were extracted and then dried in an oven at 60 °C temperature. From here, each sample was divided into two equal parts for the subsequent analyses that we have defined as morphological and molecular, referring to the treatment for microscopic observation and molecular sequencing, respectively.

#### Morphological analysis

The initial sample volumes ranged from 100 to 400 mL. After allowing sedimentation for 24 h, during which the suspended material settled to the bottom of the recipient, the supernatant was carefully removed. From the remaining sediment, 10 mL of the unaspirated material was pipetted per sample and transferred into tubes. The samples were centrifuged at 5000 rpm for 5 mins. Following centrifugation, the supernatant was discarded, and 5 mL of hydrogen peroxide was added to the pellet. Once homogenized, the samples were transferred to test tubes for processing following the recommendations of the “Guidance for the routine sampling and preparation of benthic diatoms from rivers and lakes” [41]. This process includes oxidation with hydrogen peroxide; in our case, we added 5 mL to eliminate organic matter over 12 h in a water or sand bath. After allowing the samples to settle for 12 h, a few drops of hydrochloric acid were added to remove carbonates. The acid was then removed following two washes with distilled water. As this is a quantitative analysis, the sedimented frustules were diluted in 5 mL of distilled water after the second wash. If the samples contained excessive salt, they were further diluted at a 1:10 ratio. Finally, 1 mL of the sample was placed on coverslips for fixation with high-refractive synthetic resin (Naphrax®) on a hot plate.

Finally, the analysis is carried out using a bright-field optical microscope (Olympus BX60, DIC, UPlanApo VC 100 objective; × 1000 magnification equipped with a 9Mp Optika camera). The diatom frustules found were photographed at × 1000. We recommend counting a minimum of 400 valves when possible, as usual in diatom studies.

#### Molecular analysis

Samples were centrifuged at 4000 rpm for 5 mins at 4 °C, using at least 200 mL of each sample. The supernatant was discarded, and the process continued from the pellet.

DNA extraction was performed using the Power Soil DNA Isolation® kit (Mo Bio Laboratories, Carlsbad, CA, USA), following the manufacturer's instructions. The *rbc*L marker was amplified by PCR using primers proposed in previous studies [42,43], a equimolar mix of five primers, three forward Diat_rbcL_708F_1 (AGGTGAAGTAAAAGGTTCWTACTTAAA), Diat_rbcL_708F_2 (AGGTGAAGTTAAAGGTTCWTAYTTAAA), and Diat_rbcL_708F_3 (AGGTGAAACTAAAGGTTCWTACTTAAA), and two reverse Diat_rbcL_R3_1 (CCTTCTAATTTACCWACWACTG) and Diat_rbcL_R3_2 (CCTTCTAATTTACCWACAACAG), including Illumina P5 (CTTTCCCTACACGACGCTCTTCCGATCT) and P7 (GGAGTTCAGACGTGTGCTCTTCCGATC) adapters.

For each DNA sample, three PCR replicates were performed using 1 μL of extracted DNA in a mixture (final volume of 25 µL) containing 0.5 µL of Phire® Hot Start II DNA Polymerase enzyme, 5 μL of Buffer 5X, 2 μL of dNTP mix (2 mM each), 0.25 μM of each primer, and 14.5 μL of nuclease-free water. PCR conditions included an initial denaturation step at 94 °C for 4 min, followed by 40 cycles of denaturation at 94 °C for 30 s, annealing at 55 °C for 30 s, and extension at 68 °C for 30 s, with a final extension step at 68 °C for 10 min. After PCR, rbcL gene amplification was evaluated using 1.5% agarose gel electrophoresis stained with ethidium bromide and visualized under ultraviolet light. DNA metabarcoding libraries and sequencing were conducted by AllGenetics & Biology SL (A Coruña, Spain).

The PCR2 reaction was carried out in a final volume of 25 µL, containing 2.5 µL of DNA from the received PCR products, 1 µM of primers with dual indices, 6.5 µL of Supreme NZYTaq 2x Green Master Mix (NZYTech), and ultrapure water up to 25 µL. The reaction mixture was incubated as follows: initial denaturation at 95 °C for 5 min, followed by 5 cycles of 95 °C for 30 s, 60 °C for 45 s, 72 °C for 45 s, and a final extension at 72 °C for 7 min. A negative control (BPCR) containing no DNA was included in each PCR round to check for contamination during library preparation. Libraries were run on a 2% agarose gel stained with GreenSafe (NZYTech), and images were captured under UV light to verify library size. Libraries were purified using Mag-Bind® RXNPure Plus magnetic beads (Omega Biotek), following the manufacturer's instructions. Subsequently, libraries were pooled in equimolar amounts based on quantification data provided by the Qubit™ dsDNA HS assay (Thermo Fisher Scientific). Pooling was sequenced on a fraction of a MiSeq PE300 run (Illumina).

Regarding bioinformatic analysis, sequences were processed using the DADA2 package in R [44], following the workflow implemented in the diat.barcode package [45], including filtering, chimera removal, and generation of amplicon sequence variants (ASVs). Taxonomic assignment was performed using the Diat.barcode v7 reference database [46].

## Method validation

Not applicable.

## Limitations

While this study presents a standardized protocol for the analysis of epiphytic diatom assemblages on *Gelidium corneum*, several limitations should be acknowledged. Firstly, although the method was tested on samples from multiple locations along the Cantabrian coast in northwest Spain, its applicability to other macroalgae or geographic regions with differing environmental conditions remains to be validated. Secondly, the vigorous shaking technique employed to detach epiphytes may underrepresent diatoms with stronger adhesion mechanisms, despite efforts to account for this bias. Alternative approaches, such as acid digestion or mechanical scraping, though more labor-intensive, could complement this protocol in specific contexts. Additionally, while the integration of molecular techniques, such as DNA metabarcoding, enhances the resolution of taxonomic identifications, the current limitations of reference databases, particularly the incomplete coverage of marine diatom species in Diat.barcode, may affect the accuracy of species-level assignments. Furthermore, linking sequence abundance to cell counts relies heavily on assumptions related to marker gene copy numbers, and although validated for rbcL by Vasselon et al. [43], this approach may not generalize to other genetic markers or diatom assemblages. Despite these constraints, this protocol provides a robust framework for future studies and offers opportunities for refinement and adaptation to diverse ecological scenarios.

## Ethics statements

The work does not involve any ethical issues.

## Related research article

*None*

## For a published article

*None*

## Supplementary material *and/or* additional information [OPTIONAL]

*None*

## Declaration of competing interest

The authors declare that they have no known competing financial interests or personal relationships that could have appeared to influence the work reported in this paper.

## Data Availability

No data was used for the research described in the article.
